# Supplementary material for: Experiences of accessing primary care by those living with long Covid in New Zealand: A qualitative analysis
Source: PLoS One. 2025 Nov 5;20(11):e0324489. doi: 10.1371/journal.pone.0324489 (PMC12588452; doi:10.1371/journal.pone.0324489)
Supplement: S2 Appendix — (DOCX) [file pone.0324489.s002.docx]

# S2 Appendix: Study invitation

Kia ora

My name is Sarah Rhodes. My background is as a physiotherapist specialising in cardio-respiratory care. I currently work as a lecturer at the School of Physiotherapy, University of Otago. Since early in the COVID-19 pandemic my colleagues and I recognised that there was likely to be people impacted by the post viral effects of the infection.

As part of my research, I am hoping to connect with those living with long COVID, particularly those who are sometimes disadvantaged by the health system – Māori, Pasifika, disabled people, and those living rurally. I am seeking to understand their experiences of living with long COVID and access to health care, as well as their perspectives on what a good long COVID service would look like. i.e. what would support them most effectively.

These discussions would be run via Zoom, meaning people can be anywhere in the country and do not need to be able to get somewhere to have their voice heard. The zoom space is a safe and non-judgemental one where everyone’s input is valued. Those with lived experience of long COVID are best placed to comment on these issues. It is hoped that this information will be used to in future to demonstrate the need for a long COVID service and provide some insights into what that might look like.

If you are living with long COVID and think you might like to join a zoom group to discuss your experiences of accessing health care to date and what might help you in future, please feel free to email me for more information: [sarah.rhodes@otago.ac.nz](mailto:sarah.rhodes@otago.ac.nz)

With thanks

Sarah
