# Supplementary material for: Experiences of accessing primary care by those living with long Covid in New Zealand: A qualitative analysis
Source: PLoS One. 2025 Nov 5;20(11):e0324489. doi: 10.1371/journal.pone.0324489 (PMC12588452; doi:10.1371/journal.pone.0324489)
Supplement: S3 Appendix — (DOCX) [file pone.0324489.s003.docx]

# S1 Appendix: Introduction to zoom interviews

Thank you for taking the time to attend this group discussion today. The purpose of the discussion is to explore your experiences of accessing primary health care during your long COVID journey and explore your perspectives on what a successful long COVID service might look like. I have a few discussion questions to guide the conversation.

Before we start, I want to stress that this is a safe non-judgemental space. Everyone has something to offer to the discussion and all opinions are valued. I appreciate this is an emotive topic. It is important everyone is given the opportunity to express their opinions and allow others to express theirs.

As indicated on the consent form, the session will be recorded. The recording will be stored securely for a brief period until an automatic transcript has been generated, after which it will be deleted. All data will remain anonymous. If you would prefer not to participate, you can leave the meeting at any time. If you wish to participate, but would prefer to keep your video turned off, please feel free to do so.

# S2 Appendix: Discussion questions

1. *What is the impact of long Covid on you and/or those around you?*
2. *What services/resources are you aware of for those with long Covid?*
3. *What has helped you with your long Covid so far?*
4. *What has been unhelpful/difficult with your long Covid so far?*
5. *Are there any barriers/facilitators to your care and recovery?*

# S3 Appendix: Codes and quotes for each theme

| Theme 1: gaslighting and validation | |
| --- | --- |
| Lack of respect/prejudice | My doctor’s not a big believer in not contributing to society. [Participant 12]  …because mainly it happens towards women, and unfortunately, it’s in that basket of male dominance and medicine, and there’s a load of paradigms around that,…,it’s linked to hysteria and depression and anxiety and a whole lot of stuff that I don’t have, you know, pretty outrageous stuff to think that’s till going on in the twenty-first century. [Participant 1]  And it’s just not listening. I would say, basic human courtesy and respect. And I don’t mean to have a hard time at the staff but, some were great, but some were really shit. If you don’t know what’s going on, say it, and don’t try and make up some bloody thing. [Participant 1]  I’m actually a really articulate person whose normally very good at getting my needs met and giving my opinion on stuff. And you are treating me like that. [Participant 1]  Denial was everything in the Victorian era and we’ve carried it on for 2 centuries afterwards. [Participant 10] |
| Denial | What I know is that a lot of people, even when they’ve been referred to a specialist, they’ve gaslighted them as well. [Participant 15]  One of the problems for people who’ve got long covid that always makes me want to cry is that people don’t believe us. [Participant 3]  So as soon as they see the clouds come over the eyes and the little smile they immediately think ‘Oh God, here’s another one that doesn’t believe what I’m saying’. [Participant 3]  Just stop gaslighting us for the, the first thing you know, the bloody thing exists. There’s at least a hundred million , maybe more people in the world who have some form of this; just bloody drop the whole ‘it’s all in your head.’ [Participant 10]  They’ll say we funded this project and that project but it’s bollocks, because they already know long Covid is a problem overseas. We don’t need to figure out whether it exists. We know it fucking exists. [Participant 11] |
| Time wasting/lack of patience | I actually explored just about every doctor I could to find one that was, was not going to fob me off. [Participant 18]    So all those frequent visits to the doctors and asking questions and getting nowhere, has not been helpful to the doctor or me. [Participant 18]  Till about six weeks, very sympathetic. Then the helpfulness and my frequent phone calls wore off on the poor tired practice. Yeah. And yeah, that was gutting, absolutely gutting. [Participant 8]  …took 10 months to get a diagnosis, so that was pretty hellish. [Participant 9]  My problem is this, is like the fact there’s no cure and there’s no treatment is kind of irrelevant because the, the, but that is the thing that is told to us as though somehow that negates being looked after. Why does that negate being cared for? [Participant 9] |
| Unfairly labelled | …someone who, effectively, my doctor’s term would be ‘a weight on society.’ [Participant 13]  Very quickly, as soon as it became problematic, referred to a different arm, which put like a depression label sort of on top of it, which I knew it wasn’t [Participant 18]  Stop trying to put labels on people that don’t exist. [Participant 1]  Actually it’s a culture of psychologising people. It’s one of those normalised practices that are actually really damaging. IT’s not a differential diagnosis. It’s actually a fucking damaging practice. [Participant 11]  There’re so many people in the long Covid Facebook group who tell us their GPs aren’t interested, not listening, don’t believe in it. And that’s got to change, you know. [Participant 3] |
| Dismissive language | That GP said, you know, I thought you were fine last time, just completely dismissing all of my symptoms. [Participant 16]  And so, we, we back to square one, being told that there’s nothing wrong with you and go home. [Participant 15]  And people just go, ‘Oh well you’re lazy or crazy.’ [Participant 15]  I took [the long Covid handbook] to my GP and I said this is a great book and I hoped she’d say could she read it. But she just said ‘Oh, everyone’s gonna come up with a book for it and this and that. She really put me down over it. [Participant 8]  But if you just saying ‘Oh, just go home. You’ll be fine or you’ll get over it. [Participant 17]  And so my doctor’s like ‘Oh, no, we won’t do anything about that. We won’t do anything about that. [Participant 13]  We did have a temporary long covid clinic that we could access down here. My doctor wouldn’t refer me for ages. So I basically got the clinic to contact her…and afterwards she said, ‘So you learned nothing did you?’ [Participant 13] |
| Validation (you’re not batshit Crazy) | …have probably been one of the biggest supports for me in that they actually took me seriously and supported me. [Participant 13]  She’s always happy to support me with medical certificates and, and you know, validated what I say. [Participant 14]  That validation is just so important like, I went to a new medical practitioner, actually a chiropractor last week. And the outstanding thing to me was, he said, ‘Oh, okay, how does long covid affect you? And he talked about it as if it was perfectly normal to not be able to remember things. [Participant 13]  But my GP was really good. I mean he could see where I was coming from. [Participant 7]  …and the respiratory guy said, ‘ you got the really, really short straw.’ [Participant 17]  I was dreading going to see this physio. Oh, my God, what am I going to get? Am I going to walk away feeling really down? But I walked away from that feeling ‘Oh thank goodness’; you know, she was spot on. She was empathetic. [Participant 8] |

| Theme 2: Lack of support/unmet need | |
| --- | --- |
| Barriers to care in the system | Because go to the doctor and they say ‘Oh no, the waiting list is a year [to see a specialist] [Participant 13]  Assessment is framed in such a way that it doesn’t actually take into account the resourcefulness of the person and what they see as something that can move them on. [Participant 17]  Before that I was spending hours in town waiting for buses to come home from appointments, and the, the energy cost was really high. [Participant 4]  There is also a funding pool at Te Whatu Ora that’s called Long term health conditions funding pool. But we, no one’s been able to access it. [Participant 4]  I struggled a lot with work and income around long covid because they require all disabilities to have an end date and no one can give nan end date when I’m going to stop having long Covid. [Participant 4]  I am just putting my rural context on that, because there’s nothing available here for me. And even if I was to have a mobility card, there’s no taxi service you know. [Participant 1]  They said I was urgent, at that point as well. I could barely speak, actually barely speak at all, and they told me I wasn’t, that it would be at least 12 months before I saw anyone. [Participant 1]  I had to buy my own wheelchair. They were going to give me a commode. They were nice enough to offer me that at the hospital after two months. But they weren’t going to get me a wheelchair. [Participant 2]  Now, my understanding from people who have had that experience [referral to a specialist], it doesn’t work because it takes a year for them to get referred on, or they still haven’t seen the person; but that sort of system has to change. [Participant 3]  I asked for a physio referral and I got a letter I the post to today it would be a four month wait, and I thought ‘Oh for God’s sake, you know, am I going to have to pay for this too?’ [Participant 8]  Even in the private sector, the wait lists are now getting out to 6 to 8 months for private patients; let alone people who rely on the public system. [Participant 9]  When I was struggling and the doctors were struggling with the case, the workload they had; I was referred to the health coach at the practice. [Participant 18]  Our health system still sucks. Our welfare system still sucks. Our housing is not designed for people with disabilities. [Participant 6]  Your doctor has to say that you’re going to be like that for 2 years. So, I can’t access disability support because she hasn’t even once, 2 months, 3 months, or 4 months, and so the clock doesn’t start. [Participant 13] |
| Limits to existing treatment/lack of care | I can’t get any assistance. So, the doors keep closing everywhere. [Participant 18]  The nature of general practice now is not what it used to be. And when I, because I rang, rang the nurse for something,…,she didn’t even ask how I was. [Participant 17]  And in fact today I have mental health visiting because I am an older person. So, I’m going through that second layer of what is fatigue and what is depression? [Participant 18]  Unfortunately, I’m 66, so now I come under older adults, so I can’t event access things that are sensible. It’s, I have to be checked for dementia and mental health came and did a whole check on dementia and it was quite demeaning. [Participant 18]  Even people who are eligible for home help can’t get home help because of the way they fund the carers. [Participant 1]  But again she [the physio] gave me some exercises which I did but I had to top doing them because they just made me nauseous. My balance was off and they fatigued me so much. [Participant 1]  Then they weren’t sure they saw some things on the scan, and they hummed and hawed and then said ‘Oh, maybe psychological.’ Then my GP didn’t really go with that. But he didn’t really know where to go because it’s a hierarchy. [Participant 11]  Basically, I’ve been left to rot. I’ve got no support. I need some support. [Participant 11]  The support from GPs is so variable and it’s almost troubling to think that. [Participant 13]  If it’s not a physical, if you don’t have a brain injury per se but your brain Is being affected by a virus, there just seems to be this silence around it. [Participant 9]  Came home early [from surgery],…, so ended up isolated at home unable to drive for a month. No follow up arranged by hospital with district nurse. [Participant 18]  Because that’s what the GP services are meant to be doing is this sort of, but they’re just not doing it. The nurses are under the pump, the doctors are under the pump. [Participant 8] |
| Access to care declined | My GP wouldn’t give me a referral to a physiotherapist. [Participant 16]  So they rejected that, that referral from the GP, because they said we can’t see, we can’t see you because that’s not a symptom. [Participant 15]  It’s very real having to go through just being declined and declined and having an advocate trying to just, I could not access the specialists to get the diagnosis ACC wanted. [Participant 18]  ACC declined the claim. It just said exacerbation of existing conditions and that was one of the reasons it was declined. [Participant 18]  We’re waiting to see if I can, finally, 2 years on, get through for a brain scan which has been declined from neurology and now were going to ear, nose and throat. [Participant 18]  I was prescribed a month ago medical CBD oil to try and ease my symptoms, but I can’t afford it, and I can’t get WINS to pay for it unless I can prove we have tried absolutely everything. [Participant 4]  I was declined access to a dietitian. [Participant 6] |
| Limited perceived value/loss of confidence in the health system | I’m a survivalist but there’s probably nothing that will come from those appointments other than exhausting me. [Participant 18]  But there’s actually been no direct, anything directly new being offered to me for the range of symptoms I‘ve got. [Participant 18]  I mean, I’ve almost given up going to the doctors. To be honest, there’s, there’s no point. [Participant 18]  They sent some guidelines, I understood, about 12 months ago to doctors. You know, what are the protocols? I haven’t seen in my own practice that they’ve been given to assist people like me because I haven’t seen any changes at all. [Participant 18]  I could probably count on one hand how many times I’ve been able, like, been actually to the doctors in the last 15 months; there’s just nothing they, they could do. [Participant 2]  I’ve lived with long Covid for a year. I finally get to go into the clinic that has such a long wait time and when I come in there’s no change to my health plan for me. [Participant 5] |
| No practical support | I just want to be seen and given some sort of recovery plan – even if it’s just achieving something small in my recovery that would be better than the endless self-management and flares. [Participant 18]  But there’s been no support. I almost wish I’d been in a car accident because then at least I would have some support. [Participant 2]  I’ve been trying to get home help for a year. I’ve spoken to two GPs, several nurses, 2 social workers, a Te Whatu Ora provider, several MPs, couple of journalists, a lawyer specialising in disability rights and several public servants, including people who were members of the Ministry of Health and long Covid working group who are friends of mine. And despite all of that, I still don’t have any home help. So that’s an ongoing battle. [Participant 4] |
| Poor advice | And there’s still physios doing graded exercise therapy for people with long covid. [Participant 15]  Because for me personally, I’ve had doctors who use ineffective forms of tests for the orthostatic components. [Participant 5] |
| Lack of guidance/information/resources | But several months ago, when there was the possibility to get flu shots before winter, I, you know, was sort of faced with a dilemma. Do you spend the 50 bucks to get the flu shot, no knowing whether it’s a good idea or a bad idea to get it as somebody with long Covid? … And I would have appreciated some information about that when the flu shots were going round. [Participant 14]  You’ve got post-exertional malaise PEM which I, I don’t even know what the **** that is, you know, really. And then I had immunology put. ‘Oh she’s probably got POTS on top of it now. I’ve never been, you know, so it’s, I’ve been put into a basket, and without even actually checking. I, I now have all these labels put on top of me with no advice you know. [Participant 18]  Everyone goes ‘oh the poor [GP] practice, you know, the poor practice, they are so under pressure, it’s like, well stuff it actually! You know, this is where you’re meant to be coping with us. The Government has said you are responsible for this chronic illness but they haven’t got the resources to do it. [Participant 8] |
| GP doesn’t know enough/lack of GP action | But I think the basic issue is that the doctor just doesn’t know enough to be able to say something that’s helpful in terms of care. [Participant 14]  But [my GP] can’t give me advice on what I can do to manage my symptoms. [Participant 12]  So I’ve had a helpful GP but who, like others, has been supportive but unable to do anything other than be supportive in a visit in terms of empathy. So, I’m grateful for that too but to have absolutely no care available is just, you know, I think it’s just awful. [Participant 15]  My current doctor, as others have said, she’s really understanding but is not doing anything. It’s kind of like a wall. “But we don’t know long Covid, so I’m not gonna investigate any further.” [Participant 16]  I’ve had really like kind and caring GPs; they haven’t known what the f**k to do with me. [Participant 4]  What I found gutting about my GP eventually was like she’s shrug her shoulders and go ‘Well we don’t know much about it’ and it’s like, for goodness sake, they’ve had it in the world for three years. [Participant 8]  And it wasn’t anyone’s fault that they could offer me, they could only offer me what they , yeah, what they clinically knew was… [Participant 17] |

| Theme 3: Inequity of available care | |
| --- | --- |
| Variability of PC experience (some no help, some good) | You know it’s just appalling that the people running a long Covid clinic are so unfamiliar with what, what the symptoms of long Covid are. [Participant 15]  But GPs are autonomous practitioners,…, they still get to choose whether they engage with it or not, and whether they are, and that makes it so random, and has so many people like XXX being treated so appallingly. [Participant 15]  And so, you know, how do you find a good GP? [Participant 13]  Because go to the doctor and they go ‘Oh no, the waiting list is a year.’ I went to my physio, who put the information through, and I saw that person a week later because I was deemed urgent. [Participant 13]  I mean, my GP was really good, he could see where I was coming from and he just, he said I’d go along. [Participant 17]  I mean, there are good general practices, and they’re really, really busy. And you can’t get into the see the GP for 2 weeks basically. [Participant 17]  I just think of people that have got heart failure or diabetes and all, they get seen every three months, You know, they’ve got a connection to their GP or their practice nurse every three months. [Participant 17]  What I’ve had access to is amazing. I mean amazing volunteers from our Medical centre in terms of rural nursing. [Participant 1]  My GP has been amazing; don’t know if she’s going to continue to be amazing if I continue to be seen because I think chronically ill people are a little bit, you know. It’s a bit of a, they don’t really like them very much but at the moment she’s still amazing. [Participant 3]  My GP has been really incredible. The whole clinic has been. [Participant 1]  And so it was a really wrap around kind of approach to care. And I really appreciated that. And it made a big difference, because I didn’t always have to keep explaining myself either. They, they did a lot of that conversation because I wasn’t really up to communicating very much at all. [Participant 1]  In Wellington, the doctor services aren’t nearly as good. I didn’t even have that where my house is, it doesn’t really have any doctors anymore. So, I don’t have a doctor and I could probably count on one hand how many times I’ve been able, like, been actually to the doctors in the last 15 months; is just nothing they can do. [Participant 2]  It’s like so piecemeal, is the way I would describe it at the moment. You’re really lucky when you find someone good. [Participant 8]  I was very lucky to even get diagnosed with it. [Participant 5]  They almost were blocking me from accessing my GP. [Participant 8]  The two GPs I’ve had, if I advocate, they acquiesced. [Participant 9]  And so they keep this dialogue between the three of them going. And so whatever one of them was thinking or treating at the time, they would all treat me and think that way. And so I was a really appreciated that. And it made a big difference because I didn’t always have to keep explaining myself either. [Participant 1] |
| Experiences of allied health | He’s [physio] really good because he’s recording stuff,…., and I said, I said I think my rib cage is actually working better,…,he said ‘You’re halfway there.’ He’s very encouraging. [Participant 17]  He said ‘Well, I’ve heard one of my clients had got some walking poles and it’s brilliant. This person says it’s brilliant. Why don’t you try it?’ So he’s picking up on what other people are doing and offering it to someone else….It’s lived experience knowledge. [Participant 17]  Just someone mentioned that they went to physio, who is able to identify exercise intolerance. And I thought that was so revelatory; just, yeah, like, that’s what it is. That’s a wonderful term to express it. And, yeah, so obviously that fits into the diagnosis. [Participant 16]  I saw the occupational therapist once and they gave me a piece of paper on pacing, and that was it. [Participant 1]  The physio came too for a house call, which I was amazed at,…,But again she gave me some exercise which I did but I had to stop doing them because they just made me nauseous. [Participant 1]  I was able to access the hospital physiotherapist and was given more support, and aides to help my day-to-day living – a bath seat and a stool. [Participant 6]  So the dietitian, I cried my way through the whole consultation. She was amazing and she just put me straight onto Ensure. [Participant 8]  I’m paying for a psychologist, you know, for a counsellor, and I don’t really feel like she’s given me anything. I tried a HIP; she was pretty useless. [Participant 8]  And there’s still physios that are doing graded exercise therapy for people with long Covid. And if you’re going to have physio referrals, they need, physios need updating and educating as well. [Participant 15]  And I just needed someone to say, to say to me ‘ Hey, you know it’s okay that you’re bruised right down one side because when you get out of bed, you’re not even upright; you’re leaning at an angle.’ But none of that came from the GP. That all came from me taking myself to the physio. [Participant 13] |
| Inequity of access between individuals | I know someone who’s had long Covid since the she had Covid at the start of March this year; so only months. And she’s already got a POTS diagnosis. She’s already seen cardiology. She’s in the same city as me. She’s as sick as I am but she can get all of that stuff. Is still can’t even get referred for a tilt table test. It’s variation even within cities. [Participant 4]  It’s so variable from GP clinic to GP clinic. [Participant 1]  Those who are diagnosed (long Covid/fibro/chronic fatigue) still can’t get support. [Participant 6] |
| Rural limitations | I’m just putting my rural context on that because there’s nothing available here. And even if I was to have a mobility cared, there’s no taxi service you know. There’s, there’s not, nothing to use anything like that ,…, to benefit me,…, in our rural area and getting home help is really difficult to come by. [Participant 1] |
| Public versus private/costs | He put me onto CBD and I paid a fortune for that [Participant 8]  So I pay privately for neuro psych assessments at that stage where they thought speech help because it’s affected my language part of the brain. [Participant 11]  I’m self-funding quite a bit of it, or my brother is. [Participant 13]  My GP offered for me to go to a private respiratory specialist and pay hundreds of dollars in order to be able to get into this long Covid clinic…[Participant 15] |

| Theme 4: lack of upskilling of health care staff | |
| --- | --- |
| Lack of support for GPs/nurses | She [the HIP] doesn’t know what to do. I feel so sorry for her. She’s I think, she’s overwhelmed. [Participant 18]  I know there’s a lot of chronic disease stuff happens in primary health care but how on earth can they not have more support for primary health trying to do it? [Participant 8] |
| Lack of appropriate knowledge amongst health professionals | But the basic issue is that the doctor just doesn’t know enough to be able to say something that’s helpful in terms of care. [Participant 14]  So, I’ve had a helpful GP but who, like others, has been supportive but unable to do anything other than be supportive in a visit in terms of empathy. So, I’m grateful for that but to have absolutely no care available is, you know, I think it’s just, it’s, it’s awful. [Participant 15]  And there are still physios doing graded exercise therapy for people with long Covid. And, if you’re going to have physio referrals, they need, physios need updating and educating as well. [Participant 15]  With my current doctor,…, she’s really understanding but is not doing anything. It’s kind of like this wall: but we don’t know long Covid, so I’m not gonna investigate any further. [Participant 16]  There should be a baseline level of standardised knowledge across GPs. We’ve got enough information and data coming in to kind of recognise what long Covid is. [Participant 12]  I’ve never known a condition where a doctor doesn’t sit down, take you seriously, and perhaps suggest a plan or a way through. [Participant 18]  I think there needs to more health professional education around it. [Participant 8]  Then my GP didn’t really know where to go with that. [Participant 11]  I think there needs to be a checklist of things that you GP needs to keep doing without you advocating for it. You know, things like every three months. [Participant 9] |
| Lack of explanation | What I would love to be able to do is go to a medical professional and they could guide me through finding the right language to, to say what it is I am experiencing… [Participant 14]  You’ve got post exertional malaise – PEM – which I, I don’t even know what the f*** that is, you know, really. And then I had immunology put ‘Oh she’s probably got POTS’ on top of it now. I’ve never been, you know, so it’s, I’ve been put into a basket and without even actually checking. I have all these labels put on me but with no advice. [Participant 18] |

| Theme 5: Let down by Government | |
| --- | --- |
| Let down and angry | I don’t know if you’ve got any friends in the Ministry, but I was really, I was really disappointed when they disbanded the advisory; the long Covid advisory. [Participant 17]  I feel that New Zealand’s carrying on with momentum and I’ve been left behind. [Participant 18]  I feel totally let down by the health system; start me on that, I’ll never shut up. [Participant 11]  I have no respect for the health system. [Participant 10]  I mean, I feel totally pissed off with the Government,…, But for the Government to say, to be so silent on long Covid. I mean, it’s not just, it’s illegal. [Participant 11] |
| Lack of resourcing | I’m in Christchurch and we apparently had a long, you know, supposedly the only Te Whatu Ora funded long Covid clinic in the country. But it was pretty pathetic, really. It was only a physio and there was absolutely no medical input at all. [Participant 15]  The Government has said you [primary care] are responsible for this chronic illness, but they haven’t got the resources to do it. [Participant 8] |
| Systems failure | So it’s greatly ironic that I’m actually here today and extremely angry because everything I planned for [in terms of policy] has been ignored by the public policy system. [Participant 10] |
| Lack of guidance | I just haven’t seen any guidance from the Ministry of Health. [Participant 14]  There were a few regions who had pilots but they were funded on a trial basis by Te Whatu Ora based on the recommendations of the long Covid working group. But they didn’t include all the recommendations of the long Covid working group because the group also recommended that we have access to, for instance, home help and domestic support. [Participant 4] |

| Theme 6: Self-advocacy and its cost | |
| --- | --- |
| The need to self-advocate | Because of the barriers like, I find myself having to really advocate for more extra time and stuff. [Participant 12]  I’ve found some helpful stuff online but it’s all up to you. We, we search and we find. [Participant 15]  I think that, probably with me, I was bolshy enough and, and then annoying enough to get what I wanted. [Participant 17]  So, you’re really having to advocate quite strongly for yourself in that space. [Participant 18]  I’ve actually, you know, in a very businesslike way, sat down in front of multiple doctors. I managed to get back into the health system for the referrals that I needed, but that, of course means more work for me. [Participant 18]  But in the meantime, I felt like I was, I’ve been all along, I felt like I’ve had to manage as a nurse case; manage my own case. [Participant 8]  I’m left to hunt out researchers for brain problems myself. [Participant 11]  So I have to get my notes and f***ing point it out to the doctor; just f***ing irritating [Participant 11]  When I was getting really desperate and needed to see somebody for my mental health, I ended up ringing the PHO. [Participant 8]  My doctor wouldn’t refer me for ages. So I basically got the clinic to contact her. I’d given them permission to look at my medical records. So I went over her head to do it. [Participant 12] |
| The impact of self-advocacy | But, you know, to advocate by yourself you know, as, so that day was just like, I was, you know, I was sick for the rest of the week. [Participant 18]  You know, like all that work, all that advocacy that I have to do, all of that in order to get them to listen is infuriating. [Participant 9] |
| Sharing advice online (patient support) | So people are now looking to others who’ve had shared experiences.,…, we’re trying to help ourselves by forming this network,…,And we’re trying to give ourselves the support and information and the knowledge because we’re not getting that from the medical… [Participant 12] |
| Proactive | And I said, what do you need for a referral to optimise the chance of it, it being accepted? [Participant 15]  And then so about 2 months ago I was seeing people talking about Breathing Works. And I thought, right, I’m going to my GP. ‘I want you to send me everything that you’ve got on Breathing Works because I need an appointment there. [Participant 17]  So I thought, well, I belong to the gym. I’ll go and see them and I’ll see if they can give me some sitting down exercises. [Participant 17]  So I made sure there was a paper trail of what I have gone into and what my feelings are, not in a complaint way, in a very factual way, to be scanned and put into my notes. [Participant 18]  It’s just if they have a responsibility to do something and they’re not, I will, I will remind them that they, they do have a responsibility. [Participant 18]  So, I’d go to my doctor initially every two weeks, you should give me a medical certificate. [Participant 3]  I think we’ve all had to do a lot of investigation into stuff because when there is no one else that’s had it and you know with my GP I was constantly sending her links; please read this, you know. [Participant 1]  They’ve just published a lovely article from the Mental Health Foundation, I think it is, and I gave it to our health man at work. He’s constantly being bombarded by me with information… [Participant 3]  I got that by advocating for cognitive testing that my doctor said yes to [Participant 9]  And basically, we’re all scurrying around arguing our own case with volunteers. [Participant 11]  And I starts, actually, it was almost like a business meeting, typing out what I’d come for and what I wanted to cover, to make sure in my 10 minutes I’d covered it. [Participant 18] |
| Finding own solutions to managing | I’ve got to read and learn myself about how I could then apply it to my schedule. But there is a huge energy cost to that. [Participant 12]  Essentially, we all seem to be doing our own individual research and then pooling our experiences with other people. [Participant 12]  I am also a member of Complex Chronic Illness Support, a chronic fatigue charity who have educated me about my conditions and are the ones I currently give credit to for my first big leap in recovery. [Participant 7]  They can’t do that [their job] anymore but they’re able to do something underneath it. But they’ve, they’ve worked that out for themselves you know. [Participant 9]  Because to make that happen, because I cannot sit here forever waiting for something to change without any support from doctors or anybody. I have to, I have to find something myself. [Participant 18] |
| Sourcing resources | I found the videos from MS support really helpful. They were great and wonderful for my parents to get on the same page. [Participant 16]  These resources are all ones I had to research for myself. [Participant 7]  So I thought, Oh I’ll go on the Internet and start searching. So I went on the UK sites and ta da! There was one, and there was Suzy Bolt who does, yeah, and has had some really good, good discussions and things. And there was a guy, Gez Medinger, yeah. And he had contacted a lot do really high people, you know, like, and you probably know all about it. So, he had some really good podcasts on. And I thought, well, this is where I’m getting my information. [Participant 17] |

| Theme 7: Throwing money at it | |
| --- | --- |
| Costs of accessing treatment | I’d wasted my hundreds of dollars, and we know that lot of the CT scans and spirometry come up normal anyway [Participant 15]  I’m self-funding quite a bit of it, or my brother is [Participant 13]  So, my doctor’s insisting I pay for double appointments [Participant 18]  But yeah, unless you’re paying privately, that’s pretty much all there is in Wellington. [Participant 2]  So, I went private, and I was one of the lucky ones; I had the financial resources to make some of it happen. [Participant 8]  I was going to get cognitive testing privately, but I couldn’t get my health insurance to go for it. So, it was going to cost $5000 or something. [Participant 9] |
| Trying anything | I read a piece by Ed Young in the Atlantic, where he just mentioned in passing that eating fibre diet, rich in fibre could affect people’s recovery from long Covid, …, I have no idea whether that’s good medical advice or not. [Participant 14]  Cause I went through another part of looking about your vagus nerve and trying to sort that out and having cold, having a cold bit at the end of your shower. [Participant 17]  She’s suggesting crystals and other things. [Participant 18]  I have had to fork out a lot for supplements and stuff. [Participant 1]  I’ve tried the alternative, you know. Hypobaric chambers, osteopaths, various different supplements, acupuncture, just in desperation to do something. [Participant 2]  I’ve tried over 20 different forms of treatment and therapy. [Participant 7]  Well, I’ve never been an alternative health sort of person, and it was really hard for me to go onto CBD oil. I cried the night I was going to start taking it. [Participant 8] |
| Paying out but not getting needs met | I’ve got mental health issues. I need a referral cause I’m currently paying for a psychologist, you know, for a counsellor, and I don’t really feel like she’s given me anything. [Participant 8]  You can spend an awful lot of money on alternative treatments…There’s lots of things you could spend your money on and you’d still be going, I’m not sure if that’s helping. [Participant 8]  So she told me the name of the expert, …, so I made a private appointment to see him at $405. Just about killed me. But anyway, and he had nothing to offer. He had nothing to offer. [Participant 3] |
| Risk of exploitation (vulnerable) | Online it is clear that desperate souls are open to being taken advantage of by alternative practitioners – our care should be clear in mainstream medicine so we are not open to this! [Participant 18] |

# S4 Appendix: CONSOLIDATED CRITERIA FOR REPORTING QUALITATIVE RESEARCH COREQ Guidelines

A checklist of items that should be included in reports of qualitative research. You must report the page number in your manuscript where you consider each of the items listed in this checklist. If you have not included this information, either revise your manuscript accordingly before submitting or note N/A.

| **Topic** | **Item No.** | **Guide Questions/Description** | **Reported on Page No.** |
| --- | --- | --- | --- |
| **Domain 1: Research team and reflexivity** |  |  |  |
| *Personal characteristics* |  |  |  |
| Interviewer/facilitator | 1 | Which author/s conducted the interview or focus group? | 4 |
| Credentials | 2 | What were the researcher’s credentials? E.g. PhD, MD | 4 |
| Occupation | 3 | What was their occupation at the time of the study? | 4 |
| Gender | 4 | Was the researcher male or female? | 4 |
| Experience and training | 5 | What experience or training did the researcher have? | 4 |
| *Relationship with participants* |  |  |  |
| Relationship established | 6 | Was a relationship established prior to study commencement? | 4 |
| Participant knowledge of the interviewer | 7 | What did the participants know about the researcher? e.g. personal goals, reasons for doing the research | 4 |
| Interviewer characteristics | 8 | What characteristics were reported about the inter viewer/facilitator? e.g. Bias, assumptions, reasons and interests in the research topic | 4 |
| **Domain 2: Study design** |  |  |  |
| *Theoretical framework* |  |  |  |
| Methodological orientation and Theory | 9 | What methodological orientation was stated to underpin the study? e.g.  grounded theory, discourse analysis, ethnography, phenomenology, content analysis | 3 |
| *Participant selection* |  |  |  |
| Sampling | 10 | How were participants selected? e.g. purposive, convenience, consecutive, snowball | 3 |
| Method of approach | 11 | How were participants approached? e.g. face-to-face, telephone, mail, email | 3 |
| Sample size | 12 | How many participants were in the study? | 6 |
| Non-participation | 13 | How many people refused to participate or dropped out? Reasons? | None |
| *Setting* |  |  |  |
| Setting of data collection | 14 | Where was the data collected? e.g. home, clinic, workplace | 4 |
| Presence of nonparticipants | 15 | Was anyone else present besides the participants and researchers? | N/A |
| Description of sample | 16 | What are the important characteristics of the sample? e.g. demographic data, date | 6 |
| *Data collection* |  |  |  |
| Interview guide | 17 | Were questions, prompts, guides provided by the authors? Was it pilot tested? | 3 |
| Repeat interviews | 18 | Were repeat inter views carried out? If yes, how many? | N/A |
| Audio/visual recording | 19 | Did the research use audio or visual recording to collect the data? | 3 |
| Field notes | 20 | Were field notes made during and/or after the interview or focus group? | N/A |
| Duration | 21 | What was the duration of the inter views or focus group? | 3 |
| Data saturation | 22 | Was data saturation discussed? | No |
| Transcripts returned | 23 | Were transcripts returned to participants for comment and/or correction? | 5 |
|  |  |  |  |
| **Domain 3: analysis and findings** |  |  |  |
| *Data analysis* |  |  |  |
| Number of data coders | 24 | How many data coders coded the data? | 5 |
| Description of the coding tree | 25 | Did authors provide a description of the coding tree? | No |
| Derivation of themes | 26 | Were themes identified in advance or derived from the data? | 5 |
| Software | 27 | What software, if applicable, was used to manage the data? | N/A |
| Participant checking | 28 | Did participants provide feedback on the findings? | N/A |
| *Reporting* |  |  |  |
| Quotations presented | 29 | Were participant quotations presented to illustrate the themes/findings?  Was each quotation identified? e.g. participant number | 6-15 |
| Data and findings consistent | 30 | Was there consistency between the data presented and the findings? | 6-15 |
| Clarity of major themes | 31 | Were major themes clearly presented in the findings? | 6-15 |
| Clarity of minor themes | 32 | Is there a description of diverse cases or discussion of minor themes? | N/A |

Developed from: Tong A, Sainsbury P, Craig J. Consolidated criteria for reporting qualitative research (COREQ): a 32-item checklist for interviews and focus groups. *International Journal for Quality in Health Care*. 2007. Volume 19, Number 6: pp. 349 – 357
