# Supplementary material for: Experiences of accessing primary care by those living with long Covid in New Zealand: A qualitative analysis
Source: PLoS One. 2025 Nov 5;20(11):e0324489. doi: 10.1371/journal.pone.0324489 (PMC12588452; doi:10.1371/journal.pone.0324489)
Supplement: S4 Appendix — (DOCX) [file pone.0324489.s004.docx]

# S4 Appendix: Discussion questions

1. *What is the impact of long Covid on you and/or those around you?*
2. *What services/resources are you aware of for those with long Covid?*
3. *What has helped you with your long Covid so far?*
4. *What has been unhelpful/difficult with your long Covid so far?*
5. *Are there any barriers/facilitators to your care and recovery?*
