# Supplementary material for: Experiences of accessing primary care by those living with long Covid in New Zealand: A qualitative analysis
Source: PLoS One. 2025 Nov 5;20(11):e0324489. doi: 10.1371/journal.pone.0324489 (PMC12588452; doi:10.1371/journal.pone.0324489)
Supplement: S6 Appendix — (DOCX) [file pone.0324489.s006.docx]

| Themes | Theme description | Codes |
| --- | --- | --- |
| Gaslighting and validation | This theme captures experiences where individuals feel dismissed, disrespected, or unfairly labelled by healthcare providers. It highlights the emotional toll of being invalidated through dismissive language or time wasting, as well as the crucial role that genuine validation plays in making patients feel heard and respected. | Lack of respect |
|  |  | Time wasting |
|  |  | Unfairly labelled |
|  |  | Dismissive language |
|  |  | Validation |
| Lack of support/unmet need | This theme reflects the range of barriers patients face in accessing adequate care. It includes frustrations with limited treatment options, poor guidance, and a lack of practical support. It also encompasses patients’ loss of trust in the healthcare system due insufficient knowledge from primary care providers. | Barriers to care |
|  |  | Limits to existing treatment |
|  |  | No change in care over time |
|  |  | Access to care declined |
|  |  | Limited value |
|  |  | Need for practical support |
|  |  | Poor advice |
|  |  | Lack of guidance |
|  |  | GP doesn’t know enough |
|  |  | Loss of trust in system (nothing will come of it) |
| Inequity of available care | This theme addresses disparities in healthcare experiences, focusing on variability between practitioners, geographical limitations (rural v urban), and differences in costs of care between public and private sectors. | Variability of GP experience (some no help, some good) |
|  |  | Duty of care not being met |
|  |  | Rural limitations |
|  |  | Public v. private |
| Lack of upskilling of PC staff | This theme identifies gaps in education and support for primary care providers like GPs and nurses. It highlights the need for improved training and better communication so that staff can adequately support patients. | Lack of support for GPs/nurses |
|  |  | Need for education |
|  |  | Lack of explanation |
| Let down by Govt | This theme identifies patients’ feelings of anger and frustration at government, and the perceived lack of support and funding necessary for long Covid healthcare services and policies. | Anger |
|  |  | Lack of resourcing |
|  |  | Lack of guidance |
| Self-advocacy and its cost | This theme explores the burden on patients to advocate for their own care, including the emotional and practical costs involved. It also highlights the positive and proactive side of self-advocacy, such as sharing advice online and building patient support networks. | The need to self-advocate |
|  |  | Sharing advice online (patient support) |
|  |  | Proactive |
| Throwing money at it | This theme reflects financial struggles related to healthcare, where patients pay for treatments that do not meet their needs. It also captures concerns about exploitation and vulnerability, highlighting the frustration of spending money on non-evidence-based treatments. | Costs of accessing treatment |
|  |  | Paying out but not getting needs met |
|  |  | Exploitation |
|  |  | Risk of exploitation (vulnerable) |
