# Supplementary material for: Experiences of accessing primary care by those living with long Covid in New Zealand: A qualitative analysis
Source: PLoS One. 2025 Nov 5;20(11):e0324489. doi: 10.1371/journal.pone.0324489 (PMC12588452; doi:10.1371/journal.pone.0324489)
Supplement: Attachment — Submitted filename: Plos One_Experiences living with long Covid NZ.docx [file pone.0324489.s007.docx]

**"Experiences of accessing primary care by those living with long Covid in New Zealand"**

This manuscript provides a valuable qualitative inquiry into the lived experiences of individuals with long Covid in New Zealand, focusing on an important gap in NZ’s primary care system. Below are the comments to be addressed:

1. Convenience sampling from an online support group may introduce selection bias. Individuals who are active in such forums may be more health-literate or or having more severe symptoms than the general long Covid population.
2. Majority of the participants holding postgraduate degrees, limiting representativeness. It has been listed as one of the limitations of the current study. The authors shall put more emphasis on how this may influence self-advocacy and health system navigation is warranted.
3. The absence of Asian participants raises questions about inclusiveness.
4. The low number of male participants (3 out of 18) may also skew findings.
5. There is limited discussion on whether thematic saturation was reached.
6. The study is over-reliant on subjective interpretation. While narrative inquiry values lived experience, stronger triangulation—such as linking findings to provider perspectives or health system data—would enrich the analysis. The current presentation occasionally risks anecdotal generalisation.
7. Some participant quotes use emotive or colloquial expressions (e.g., “they haven’t known what the ***k to do with me”), which, while authentic, shall be better paraphrased for clarity and professionalism in a peer-reviewed academic journal.
8. The term “gaslighting” is used throughout without clear definition.
9. Implementation science frameworks (e.g., CFIR, RE-AIM) can be used to structure questions and recommendations.
