# Supplementary material for: Experiences of accessing primary care by those living with long Covid in New Zealand: A qualitative analysis
Source: PLoS One. 2025 Nov 5;20(11):e0324489. doi: 10.1371/journal.pone.0324489 (PMC12588452; doi:10.1371/journal.pone.0324489)
Supplement: Attachment — Submitted filename: Response to Reviewers.docx [file pone.0324489.s011.docx]

Thank you to the reviewers for taking the time to scrutinise our manuscript and provide valuable feedback to strengthen it. It is much appreciated.

Please note the page and line references below pertain to the clean copy of the revised manuscript.

| Reviewer 1 comments | Authors’ response |
| --- | --- |
| Convenience sampling from an online support group may introduce selection bias. Individuals who are active in such forums may be more health-literate or having more severe symptoms than the general long Covid population. | This form of sampling does indeed introduce the possibility of selection bias. This has been acknowledged as a limitation of the study (page.22, line 447) |
| Majority of the participants holding postgraduate degrees, limiting representativeness. It has been listed as one of the limitations of the current study. The authors shall put more emphasis on how this may influence self-advocacy and health system navigation is warranted. | Those holding postgraduate degrees are likely to have higher health literacy and be more likely to engage with primary care services, so are not representative of the wider NZ cohort living with long Covid. This has been acknowledged in the limitations section (page 22, line 451) |
| The absence of Asian participants raises questions about inclusiveness. | This is a valid point. We are unable to comment on the diversity of members in the FaceBook forum but acknowledge the absence of Asian participants may reflect a lack of inclusivity. We acknowledge this lack of representation also affects how representative our sample is. (page 22, line 456) |
| The low number of male participants (3 out of 18) may also skew findings. | This may have skewed the findings and we acknowledge this in the limitations section. However, we also note that females appear to be at higher risk of long Covid than males. (page 22, lines 457 and 460) |
| There is limited discussion on whether thematic saturation was reached. | Thematic saturation is a hotly debated topic in qualitative analysis. We based our recruitment numbers on the concept of information power and considered a sample of 15 or greater would be sufficient to obtain the depth of data to answer the research question, given that in-depth interviews and discussion were the main means of data collection. (page 7, line 133) |
| The study is over-reliant on subjective interpretation. While narrative inquiry values lived experience, stronger triangulation—such as linking findings to provider perspectives or health system data—would enrich the analysis. The current presentation occasionally risks anecdotal generalisation. | Thank you for this constructive feedback. We have revised the discussion to include NZ health information, experiences of primary care providers and media perspectives. These have been added throughout the manuscript where relevant, to provide better triangulation of the points made. |
| Some participant quotes use emotive or colloquial expressions (e.g., “they haven’t known what the ***k to do with me”), which, while authentic, shall be better paraphrased for clarity and professionalism in a peer-reviewed academic journal. | This has been addressed and such quotes amended by eliminating the emotive language of the statement. The example given here has been revised to “they haven’t known what to do with me” |
| The term “gaslighting” is used throughout without clear definition. | A definition of gaslighting has been provided and referenced. (page 11, line 191) |
| Implementation science frameworks (e.g., CFIR, RE-AIM) can be used to structure questions and recommendations. | This is a useful observation. The RE-AIM framework will be considered in the development of a further document one data has been gathered from other stakeholders, such a GPs. |
| Reviewer 2 comments |  |
| **Methods Section** |  |
| Please include the reference for the COREQ guidelines used (lines 79-81) | The reference for this has been added. (page 5, line 77) |
| Please include reference to supplementary S4 file (lines 79-81) | This reference has been added. |
| Please refer to supplementary files using PLOS One guidelines (e.g., S1 File). (Line 117, 118, and 143) | The supplementary files have been renamed S1 Appendix etc as per the PLOS guidelines. |
| Who were the researchers that conducted data familiarisation, made notes and developed the codes? Is it SR AND CD? Please include the initials to clarify. (line 139) | This has been clarified and both SR and CD’s initials have been added for clarity. (page 8, line 162) |
| Please discuss how the study address quality assurance and qualitative research trustworthiness using known or other criteria, such as those by Lincoln and Guba on confirmability, transferability, credibility, dependability, triangulation etc. | The strategies employed to enhance trustworthiness have been outlined in the following places:  Line 104-106 – reflexivity  Line 130-133 – member checking  Line 144-146 – code-recode strategies |
| There is no comment on data saturation or information power. At what point and why were the researchers satisfied they had enough information? Please see the latest thematic analysis guide by Braun and Clarke (Braun V, Clarke V. Thematic analysis: a practical guide. London: SAGE Publications; 2021) and other resources on saturation and information power in qualitative research. | A comment has been added on the sufficiency and information power of the data. (page 7, line 131-139)  Data saturation is not a well-supported construct in Braun and Clarke’s thematic analysis approach. Ref: [Full article: To saturate or not to saturate? Questioning data saturation as a useful concept for thematic analysis and sample-size rationales](https://www.tandfonline.com/doi/full/10.1080/2159676X.2019.1704846#d1e205) |
| Please state the exclusion criteria used for study participants if any were considered. | Exclusion criteria have now been stated. (page 5, line 81) |
| Please explain how the interview guide was developed. For example, was a pilot study conducted to assess its applicability and readiness before the individual and group interviews? | The interview guide was developed by the research team and then piloted by two non-medical lay people. Feedback was obtained and tow questions were subsequently amended in response to this. (page 7, line 126) |
| It is unclear how many individuals out of the 18 participated in the individual interviews and how many in group discussions. It appears participants were invited to take part in a group or individual zoom session based on preference (lines 108-109).  Please provide more information about recruitment methods used and supply those as a supplementary file(s) for the benefit of future studies and research. | This detail has been clarified (page 9, line177).  The participants were recruited using a study invitation posted on the group FaceBook page. This is included as a supplementary file. (S2 Appendix) |
| Was there any software used during, and for data analysis? Please clarify in this section if that was the case. I appreciate there was a NO tick in Appendix 4, however, this still needs to be mentioned somewhere in the methods section. Was it all manual or did it involve excel or other programs? | There was no software such as NVivo used in the data analysis. Data excerpts were input into an Excel spreadsheet to aid the code grouping and re-grouping process to facilitate the generation of themes. (page 9, line 165) |
| Can the authors explain which thematic analysis style was used to analyse and code the data? Braun and Clarke’s method is called reflexive thematic analysis. The article only refers to thematic analysis. There are many types of thematic analysis approaches. For example, please see discussion in their book (Braun V, Clarke V. Thematic analysis: a practical guide. London: SAGE Publications; 2021). | Reflexive thematic analysis was used in the style of Braun and Clarke. This was an oversight on our part to not specify this in the original document. This has now been rectified. (page 8, line 157) |
| There are no definitions provided for each of the 7 themes to show what each finding mean. Please refer to Bruan and Clarke’s book mentioned above and other resources on defining and naming themes (lines 143 and supplementary file 3). | Descriptions for each of the themes have now been included in the supplementary file S6 Appendix. |
| **Results and discussion section** |  |
| Can you please provide more information about your definition of urban, semi-rural and rural areas in relation the NZ context? (Table 1) | This definition was based on the Geographical Classification of Health definitions. For clarity, Table 1 has been modified to reflect the full range of codes and a brief description of urban and rural classifications added. |
| Related to the above, it would be useful to include some information about where participants lived (indicated by suburbs or another way). This can give a perspective about primary care provision and whether participant experiences were nation-wide versus concentrated in certain areas. | Information about where participants lived by region has been added to Table 1. |
| In general, I noticed, at times, a lack of reference to NZ studies, news article or other supporting information related to COVID-19 and sufferers of long COVID. For example, are the studies used to discuss the results international? If so can you indicate where they were conducted? | We have revised various parts of the discussion to better reflect the NZ landscape, including reference to NZ Government reports and a few NZ studies (there are not that many). Where we have relied on overseas studies to support a point, we have reported which country the research was conducted in. |
| Have there been any discussions in the news, by primary health care or allied health professional bodies/associations or other government or non-government entities regarding long COVID, patients with long COVID, payment access, guidance development, professional training etc to refer to and weave into the discussion that is relevant to NZ or by international organisations (e.g. WHO or others) that apply to NZ? | We have now included reference to a number of NZ media reports and press releases by professional organisations throughout the discussion section to provide a broader perspective on what is happening in NZ and elsewhere. |
| **Strengths and limitations** |  |
| There is no reflection on the strengths of qualitative research in comparison with other methods. What does qualitative research methods offer and did these help participants and achieve the study aim? | The method on qualitative research has been reflected on and the benefits highlighted. (page 21, line 435-439) |
| Were there any benefits or barriers to the group sessions? | Both the benefits and barriers have been highlighted in the strengths and limitations section (page 21-22, line 442-446) |
| Did the different participation methods offered help participants and their recruitment? Was this useful, especially in terms of working around their circumstances and health symptoms (e.g., fatigue)? | This point has been acknowledged in the strengths and limitations section (line 439-442) |
| **Conclusion and recommendations** |  |
| A lot of the underlying comments were about the amount of money sufferers had been enduring. However, the conclusion and some parts of the discussion conceded that government funding is unlikely. Why is this assumed and shouldn’t this be a clear recommendation, especially given the amount of money that patients are having to spend and differences in their symptoms, needs and socio-economic backgrounds? | Thank you for this perspective. We agree this is a responsibility of Government. The conclusion has been largely revised to better reflect why Government should take more responsibility for supporting those living with long Covid. |
| I would suggest including recommendations in a separate section or at the end of the conclusion section with clear bullet points. These could be divided into areas: those for government, those for primary health care providers, those for policy-making, funding etc. At present, recommendations appear to be lacking and scattered across the results/discussion section.  This also links back to my earlier comment about the lack of weaving of NZ-specific discussion points which may help reach some of these recommendations. | Thank you for this suggestion. We have added a recommendations section after the conclusion, which links to some of the outcomes from the data. |
| Having just an awareness campaign and better utilisation of allied healthcare is useful but are they sufficient given the 7 themes and several sub-themes discussing different systemic, cultural and other issues participants mentioned?  For example, there is a clear oversight regarding the role of medical associations, staff training, having paid vs volunteer services on offer, lack of awareness, inequity in access, having joint-up services, availability of private vs public funded clinics and affordability by patients. Not mentioning any of this risk simplifying the issue and is not reflective of the discussion points raised by participants. Also, the lack of mention of government role or the role of primary health care providers or other actors risk releasing them from their responsibilities, as a result perpetuating the same systemic issues. | We have revised the recommendations and made them more explicit as per your previous point. It is hoped this provides greater emphasis on the responsibility of Government and primary care in the management of long Covid in NZ. |
| Are there any conclusions that could be relevant to the international community?  For example, any points of similarities or differences noticed, including handling of patient cases or government funding, guidance provision, private versus public sector health care reliance that may be worth highlighting to show global response, lessons learnt or perhaps the lack of response? | We have attempted to highlight points of difference, particularly in terms of funding, in other countries such as Australia, the US and UK. It is difficult to articulate an overall ‘global response’ as there appears to be plenty of international variation. |
| Is there a global crisis of care provision towards long COVID sufferers? Has the world left them behind? | Given the data from overseas, there does not appear to be a global crisis. People in NZ are definitely being left behind though. |
| Is there a need for recognition globally, especially the estimated number of long-COVID sufferers? | As per the previous comment, there is global recognition but not necessarily global responsibility. Long Covid is being recognised by governments in briefing papers and COVID inquiries but that doesn’t always translate to action. |
| **General notes** |  |
| Please fix all in-text referencing of the manuscript to match PLOS One style. For example, (1) should be [1] and (6), (7,8) should be [6-8]. | Thank you for drawing our attention to this. This has been rectified. |
| There seems to be few participant data/excerpts that fell into multiple thematic categories. Can the authors comment on and explain somewhere in the manuscript the coding process and allocation of codes to multiple themes?  Is this accepted in the thematic analysis style of Bruan and Clarke? | A comment about this as a process outcome in qualitative analysis has been added (page 9, line 168) |
| Were there any demographic differences to discuss that are worth mentioning following data analysis?  For example, was there a noticeable difference to maori and pacific people and minority participants compared to others in their answers and comments to mention in the discussion section and are there any relevant implications or recommendations from that? | The transcripts were deidentified prior to data analysis so it wasn’t possible to undertake this kind of analysis. It is therefore not possible to make any inferences or recommendations on this. |
