# Supplementary material for: Experiences of accessing primary care by those living with long Covid in New Zealand: A qualitative analysis
Source: PLoS One. 2025 Nov 5;20(11):e0324489. doi: 10.1371/journal.pone.0324489 (PMC12588452; doi:10.1371/journal.pone.0324489)
Supplement: Attachment — Submitted filename: Experience Long Covid_qualitative PLOS One.docx [file pone.0324489.s010.docx]

Thanks for the invitation to review the first revised version of this manuscript on “Experiences of accessing primary care by those living with long Covid in New Zealand: a qualitative analysis”. Most of the comments from the reviewers have been addressed satisfactorily. However, there are few questions below to be addressed.

1. There is limited reflexivity statement within the “Methods” section. The authors did not include professional background, prior experience with long Covid in this component. This omission limits readers’ ability to assess how the researchers’ positions may have influenced data collection, interpretation, and thematic analysis.
2. The limitations acknowledge selection bias and overrepresentation of highly educated participants. However, there is no reflection on how this bias may have shaped thematic dominance (e.g., emphasis on self-advocacy). This may limits the transferability to underrepresented groups, particularly Māori and Pacific communities.
3. There is misalignment between abstract conclusion and main conclusion. The abstract conclusion mentions “creative solutions for primary care,” whereas the main conclusion emphasises government investment and allied health utilisation.
4. Minorities-Māori and Pacific participants are represented. However, their voices are not distinctly analysed within the results. This may lead to the missed opportunity to discuss health inequities central to the New Zealand context.
