# Supplementary material for: Experiences of accessing primary care by those living with long Covid in New Zealand: A qualitative analysis
Source: PLoS One. 2025 Nov 5;20(11):e0324489. doi: 10.1371/journal.pone.0324489 (PMC12588452; doi:10.1371/journal.pone.0324489)
Supplement: Attachment — Submitted filename: Response_to_reviewers_auresp_2.docx [file pone.0324489.s012.docx]

Response to reviewers

Thank you to the reviewers for taking the time to review the revised version of our manuscript and provide further feedback.

Please note the page and line references below pertain to the clean copy of the revised manuscript.

| Reviewer 1 comments | Authors’ response |
| --- | --- |
| There is limited reflexivity statement within the “Methods” section. The authors did not include professional background, prior experience with long Covid in this component. This omission limits readers’ ability to assess how the researchers’ positions may have influenced data collection, interpretation, and thematic analysis. | More detail regarding the authors’ relevant professional backgrounds and previous experience with long Covid has been added to enhance understanding of how this might have influenced data collection and interpretation. (page 6, line 111-118) |
| The limitations acknowledge selection bias and overrepresentation of highly educated participants. However, there is no reflection on how this bias may have shaped thematic dominance (e.g., emphasis on self-advocacy). This may limits the transferability to underrepresented groups, particularly Māori and Pacific communities. | We have acknowledged the possibility that those who are highly educated are not reflective of the wider population, particularly Māori and Pacific Peoples, who are known to be underserved by the education system. (page 22, line 461-464) |
| There is misalignment between abstract conclusion and main conclusion. The abstract conclusion mentions “creative solutions for primary care,” whereas the main conclusion emphasises government investment and allied health utilisation. | The abstract conclusion has been amended to better align with the main conclusion, with a focus on the need for government investment and better use of the allied health workforce. (page 2, line 29-30) |
| Minorities-Māori and Pacific participants are represented. However, their voices are not distinctly analysed within the results. This may lead to the missed opportunity to discuss health inequities central to the New Zealand context. | The transcripts were deidentified prior to data analysis so it wasn’t possible to undertake separate analysis of these minority participants. This lack of opportunity to gain distinct insights from these groups has been acknowledged in the limitations section. (page 22, line 469-472) |
