# Supplementary material for: Experiences of accessing primary care by those living with long Covid in New Zealand: A qualitative analysis
Source: PLoS One. 2025 Nov 5;20(11):e0324489. doi: 10.1371/journal.pone.0324489 (PMC12588452; doi:10.1371/journal.pone.0324489)
Supplement: Attachment — Submitted filename: Reviewer comments -11082025.docx [file pone.0324489.s008.docx]

**11/08/2025 -** Thank you for the invite and opportunity to review this important study regarding long COVID-19 sufferers and their experiences in NZ from a primary healthcare access perspective. The paper included important findings on an overlooked area with potentially life changing implications for policy, practice and primary health care service delivery. I also thank the authors for a very readable and accessible manuscript. However, at its current form, there are key unaddressed points in different sections that warrant major revisions.

While the themes and findings were extensive and significant, these were let down by the lack of strong discussion points, recommendations and conclusions. Below I included further critical and general notes to address in the manuscript and offered suggestions where I could to help make the paper stronger.

**Methods Section:**

- Please include the reference for the COREQ guidelines used (lines 79-81)
- Please include reference to supplementary S4 file (lines 79-81)
- Please refer to supplementary files using PLOS One guidelines (e.g., S1 File). (Line 117, 118, and 143)
- Who were the researchers that conducted data familiarisation, made notes and developed the codes? Is it SR AND CD? Please include the initials to clarify. (line 139)
- Please discuss how the study address quality assurance and qualitative research trustworthiness using known or other criteria, such as those by Lincoln and Guba on confirmability, transferability, credibility, dependability, triangulation etc.
- There is no comment on data saturation or information power. At what point and why were the researchers satisfied they had enough information? Please see the latest thematic analysis guide by Braun and Clarke (Braun V, Clarke V. Thematic analysis: a practical guide. London: SAGE Publications; 2021) and other resources on saturation and information power in qualitative research.
- Please state the exclusion criteria used for study participants if any were considered.
- Please explain how the interview guide was developed. For example, was a pilot study conducted to assess its applicability and readiness before the individual and group interviews?
- It is unclear how many individuals out of the 18 participated in the individual interviews and how many in group discussions. It appears participants were invited to take part in a group or individual zoom session based on preference (lines 108-109).
- Please provide more information about recruitment methods used and supply those as a supplementary file(s) for the benefit of future studies and research.
- Was there any software used during, and for data analysis? Please clarify in this section if that was the case. I appreciate there was a NO tick in Appendix 4, however, this still needs to be mentioned somewhere in the methods section. Was it all manual or did it involve excel or other programs?
- Can the authors explain which thematic analysis style was used to analyse and code the data? Braun and Clarke’s method is called reflexive thematic analysis. The article only refers to thematic analysis. There are many types of thematic analysis approaches. For example, please see discussion in their book (Braun V, Clarke V. Thematic analysis: a practical guide. London: SAGE Publications; 2021).
- There are no definitions provided for each of the 7 themes to show what each finding mean. Please refer to Bruan and Clarke’s book mentioned above and other resources on defining and naming themes (lines 143 and supplementary file 3).

**Results and discussion section**

- Can you please provide more information about your definition of urban, semi-rural and rural areas in relation the NZ context? (Table 1)
- Related to the above, it would be useful to include some information about where participants lived (indicated by suburbs or another way). This can give a perspective about primary care provision and whether participant experiences were nation-wide versus concentrated in certain areas.
- In general, I noticed, at times, a lack of reference to NZ studies, news article or other supporting information related to COVID-19 and sufferers of long COVID. For example, are the studies used to discuss the results international? If so can you indicate where they were conducted?

Have there been any discussions in the news, by primary health care or alliwed health professional bodies/associations or other government or non-government entities regarding long COVID, patients with long COVID, payment access, guidance development, professional training etc to refer to and weave into the discussion that is relevant to NZ or by international organisations (e.g. WHO or others) that apply to NZ?

**Strengths and limitations**

- There is no reflection on the strengths of qualitative research in comparison with other methods. What does qualitative research methods offer and did these help participants and achieve the study aim?
- Were there any benefits or barriers to the group sessions?
- Did the different participation methods offered help participants and their recruitment? Was this useful, especially in terms of working around their circumstances and health symptoms (e.g., fatigue)?

**Conclusion and recommendations**

- A lot of the underlying comments were about the amount of money sufferers had been enduring. However, the conclusion and some parts of the discussion conceded that government funding is unlikely. Why is this assumed and shouldn’t this be a clear recommendation, especially given the amount of money that patients are having to spend and differences in their symptoms, needs and socio-economic backgrounds?
- I would suggest including recommendations in a separate section or at the end of the conclusion section with clear bullet points. These could be divided into areas: those for government, those for primary health care providers, those for policy-making, funding etc. At present, recommendations appear to be lacking and scattered across the results/discussion section.

This also links back to my earlier comment about the lack of weaving of NZ-specific discussion points which may help reach some of these recommendations.

- Having just an awareness campaign and better utilisation of allied healthcare is useful but are they sufficient given the 7 themes and several sub-themes discussing different systemic, cultural and other issues participants mentioned?

For example, there is a clear oversight regarding the role of medical associations, staff training, having paid vs volunteer services on offer, lack of awareness, inequity in access, having joint-up services, availability of private vs public funded clinics and affordability by patients. Not mentioning any of this risk simplifying the issue and is not reflective of the discussion points raised by participants. Also, the lack of mention of government role or the role of primary health care providers or other actors risk releasing them from their responsibilities, as a result perpetuating the same systemic issues.

- Are there any conclusions that could be relevant to the international community?

For example, any points of similarities or differences noticed, including handling of patient cases or government funding, guidance provision, private versus public sector health care reliance that may be worth highlighting to show global response, lessons learnt or perhaps the lack of response?

Is there a global crisis of care provision towards long COVID sufferers? Has the world left them behind?

Is there a need for recognition globally, especially the estimated number of long-COVID sufferers?

**General notes**

- Please fix all in-text referencing of the manuscript to match PLOS One style. For example, (1) should be [1] and (6), (7,8) should be [6-8].
- There seems to be few participant data/excerpts that fell into multiple thematic categories. Can the authors comment on and explain somewhere in the manuscript the coding process and allocation of codes to multiple themes?

Is this accepted in the thematic analysis style of Bruan and Clarke?

- Were there any demographic differences to discuss that are worth mentioning following data analysis?

For example, was there a noticeable difference to maori and pacific people and minority participants compared to others in their answers and comments to mention in the discussion section and are there any relevant implications or recommendations from that?
